# Health Tract, No. 68

**Published:** 1862-04

**Authors:** 


HEALTH TRACT, No. 68.

### THE TEETH.

Natural teeth, clean, perfect, and sound, are essential to the comeliness of any face; they not only add to the comfort and personal appearance, but contribute largely to the health of all; hence, special and scrupulous attention should be paid to them daily, from the fifth year, each tooth being minutely examined by a skillful, intelligent, and conscientious dentist every third month, up to the age of twenty-five, when they may be considered safe, with a semi-annual inspection. Avoid cold and hot food and drinks most sedulously. If a "pick" is ever employed, let it be of wood or quill. Never use a dentrifice prepared by stranger hands. Tartar on the teeth is formed by animalculæ, some of which are instantly killed by soap; others by table-salt; hence wash the teeth with a wet brush, drawn across a piece of white soap every other night, at bed time, using the salt but once a week, which, perhaps, whitens the teeth as safely and as well as any thing else. Pure sugar melts without a residue, and passes into the stomach at once, hence can not possibly hurt the teeth by its adherence to them. Heat, and cold, and acids are the things which injure the teeth on the instant of touching them. Sugar can only act perniciously in so far as by its too free use, it causes Dyspepsia. A doughnut, daily, will sooner hurt the teeth than a lump of sugar. Roast beef and canvas-back ducks, oyster-suppers, and lobster-pies, pastries, and puddings, are a thousand-fold more destructive to the teeth than pure candies, because they are the direct agents of dyspeptic disease in the masses, and disorder the stomach, generate acid gases and a liquid so sour, that when it is belched into the mouth, it has been known to take the skin off of the throat and inner lips. Much harm has been done by propagating the notion that sugar and candies are hurtful to the teeth, by drawing attention away from the general causes, such as gourmandizing hot foods, ice-cold drinks, and want of tooth and mouth cleanliness. Teeth hereditarily poor, may be kept in a good state of preservation for many years, if well watched, kept plugged in a finished style, cleaned as above, and the stomach is made to do its duty, by a temperate, active, and regular life. Great stress has been laid on the fact that a solid tooth becomes soft and pulpy if steeped in syrup for a few days, yet no apparent effect is produced on a tooth soaked for weeks in a solution of calomel, which so many claim to be a most deadly agent to the teeth. Pure sugars and candies do not injure the teeth, except indirectly, by their injudicious use, exciting acidity of stomach or dyspepsia; as will any other kind of food condiment, drink or beverage, even roast beef, brown bread, or Boston cracker, if extravagantly used. All infants and young children would die in a very few weeks, if not allowed to eat any thing containing sugar, because they need the carbon of the sugar to keep them warm; their extravagant, their insatiable fondness for every thing sweet, is a wise instinct of nature. If candies were used as desserts in winter; and fruits and berries, in their natural state, ripe, raw, and perfect in summer, to the exclusion of pies, tarts, pastries, and puddings, human life would be extended, and many dentists would have to seek other occupations.

The teeth should be washed with a stiff brush on rising, and with an old, used brush immediately after each meal, always employing lukewarm water, or holding cold water in the back part of the mouth until it is warmed. Never eat an atom after the teeth have been washed for the night. Always use the brush slowly, lest by a slip, a tooth may be scaled or broken. After meals, let the bristles of the brush be moved up and down by a twisting motion, making each one a tooth-pick. A yellowish tint to a tooth is proof of its soundness; hence do not seek to keep them of a pearly whiteness; it destroys them.

